# A decade in focus: mixed germ cell tumors with choriocarcinoma components

**DOI:** 10.1097/MS9.0000000000001314

**Published:** 2023-09-20

**Authors:** Abdullah Al-Khayal, Yasser Noureldin, Mohammad Alghafees, Areez Shafqat, Belal Nedal Sabbah, Asem H. Elhossiny, Mohamad Bakir, Mohammed Ali Omar, Tarek Ziad Arabi, Saleha Abdul Rab, Bader Alsaikhan, Reema Aldhalaan, Muhannad Alquirnas, Khalid Alrabeeah

**Affiliations:** a Department of Urology, King Abdulaziz Medical City; b College of Medicine, King Saud bin Abdulaziz University for Health Sciences; c King Abdullah International Medical Research Center; d College of Medicine, Alfaisal University, Riyadh, Saudi Arabia

**Keywords:** mixed germ cell tumours, rare tumours, Saudi Arabia, testicular cancer, tumour registries

## Abstract

**Introduction:**

This 10-year registry review aimed to investigate the clinical behaviour and outcomes of mixed germ cell tumours with choriocarcinoma components, a rare and aggressive subtype of testicular cancer, in Saudi Arabia. The study explores the demographic characteristics of affected patients, tumour profiles, and the mortality rate associated with this malignancy.

**Methods:**

Utilizing data from the Saudi Cancer Registry, the authors identified 33 cases of mixed germ cell tumours with choriocarcinoma components among 1001 testicular cancer cases recorded between 2008 and 2017. Demographic information, including age, marital status, region of residency, year of diagnosis, and 10-year survival status, were collected. Tumour factors, such as the basis of diagnosis, origin site, behaviour, grade, extension, and laterality, were also analyzed.

**Results:**

The majority of cases (78.8%) occurred in the young age group (18–45 years), and most tumours (97%) originated in normally descended testes. Grade IV (undifferentiated anaplastic) tumours and distant metastasis were present in 45.5% of patients. All cases exhibited malignant tumour behaviour. The overall mortality rate was 15%, with a mean time from diagnosis to death of 7.72 months (range: 0.5–21.5 months).

**Conclusion:**

Mixed germ cell tumours with choriocarcinoma components are rare and tend to affect younger populations. These tumours demonstrate aggressive clinical behaviour, with a significant proportion presenting with high-grade lesions and metastasis at diagnosis. The observed mortality rate underscores the poor prognosis associated with this malignancy. Our study provides essential insights into the clinical characteristics of this rare tumour subtype in the Saudi Arabian population, emphasizing the need for further research to identify prognostic factors and optimize management strategies for affected patients.

## Introduction

Highlights

- Rare and poor prognosis: Germ cell tumours mixed with choriocarcinoma components are rare, but they have a bad prognosis. These tumours have been poorly documented in the literature, highlighting the need for more research and understanding of their behaviour and outcomes.
- Young population affected: The study found that most patients with mixed germ cell tumours and choriocarcinoma were in the young age group of 18–45 years. This suggests that this type of tumour may predominantly affect younger individuals.
- Aggressive tumour characteristics: The majority of cases had grade IV undifferentiated anaplastic tumours, indicating aggressive and poorly differentiated cancer cells. Additionally, 45.5% of patients had distant metastasis at the time of diagnosis, indicating an advanced stage of the disease. These findings contribute to the poor prognosis and high mortality rate associated with these tumours.

Testicular cancers are classified into germ cell tumours (GCTs) and non-GCTs, with GCTs further divided into seminomatous (SGCTs) and non-seminomatous (NSGCTs). Despite their rarity, testicular GCTs are the most common solid tumours in men of reproductive age between 20 and 40 years old, with an incidence of ~10 in 100 000 men^1^.

Choriocarcinoma, comprised of cytotrophoblasts and syncytiotrophoblasts, is the most aggressive subtype of NSGCTs, exhibiting early hematogenous metastases^2^. The production of beta-human chorionic gonadotropin (b-HCG) hormone by choriocarcinoma is used as a marker in diagnosis, prognostic workup, formulation of appropriate treatment regimens, monitoring treatment response, and detecting potential relapse^3^. Choriocarcinomas typically present with haemorrhagic symptoms from sites of metastasis, such as the lungs, liver, and brain, often with normal-appearing testes^4^. Furthermore, clinical features such as gynaecomastia and hematogenous metastases may be observed, and serum hCG levels would typically be the highest among all germ cell tumours^5^. Due to their propensity to metastasize at the subclinical stage, choriocarcinomas have the poorest prognosis among testicular GCTs and are categorized in the poor prognosis subgroup per the International Germ Cell Cancer Collaborative Group (IGCCCG) classification system^3,6^. Additionally, risk factors associated with a poor prognosis include advanced age and an advanced tumour stage^7^. Choriocarcinomas appear grossly as a haemorrhagic nodule, with histology revealing haemorrhagic foci with viable tumour cells at the periphery^8,9^.

Pure choriocarcinoma is exceedingly rare, accounting for less than 0.3% of testicular GCTs^5,6,9^. More commonly, choriocarcinoma is seen as an element in ~8% of mixed GCTs^6,9^. Mixed GCTs with choriocarcinoma elements tend to be more aggressive and exhibit poorer outcomes than other variations of mixed GCTs^10^. Indeed, the histological presence of trophoblastic components or b-HCG elevations (signifying the presence of syncytiotrophoblasts) are recognized as poor prognostic indicators in patients with mixed GCTs^11–15^.

In Saudi Arabia, a study analyzing 1004 testicular cancer cases identified 85.2% of cases as GCTs, of which 3.6% were mixed GCTs, but their composition (choriocarcinoma or otherwise), clinical behaviour, and prognosis was not reported^16^. Our study provides a more in-depth analysis of mixed GCTs with choriocarcinoma components, exploring their demographic characteristics, clinical behaviour, mortality rates, and diagnosis-to-mortality intervals.

## Materials and methods

### Study design

This retrospective chart review included all adult patients diagnosed with mixed GCTs with choriocarcinoma components between 2008 and 2017. Data were retrieved from the Saudi Cancer Registry (SCR), which collects tumour data from all private, military, and Ministry of Health hospitals in Saudi Arabia through five regional offices (eastern, western, northern, southern, and central). The variables analyzed included the year of diagnosis, sex, age, marital status, geographical region, tumour origin site, histological subtype, tumour behaviour, grade, extent, laterality, basis of diagnosis, and survival. This work has been reported in line with the STROCSS criteria^17^.

### Statistical analysis

Data analysis was performed using the Statistical Package for the Social Sciences (SPSS) version 23.0 (IBM Corporation). Categorical variables were presented as frequencies and percentages, while continuous variables were summarized as means and SD. Patients’ ages were categorized into four groups based on the WHO’s population classification: adolescents (12–17 years), young adults (18–45 years), middle-aged (46–60 years), and senile (76–90 years).

## Results

Among the 1001 patients screened, 33 (3.3%) were diagnosed with mixed GCTs with choriocarcinoma components. The mean age of patients was 32.36 years (SD=16.17) (interquartile range=15–80) years, with the majority (*n*=26/33, 78.8%) falling into the young age group. Four patients (12.1%) were middle-aged, two (6.1%) were senile, and one (3%) was an adolescent. In terms of marital status, 42.4% (*n*=14/33) were married, 33.3% (*n*=11/33) were single, and 24.2% (*n*=8/33) were divorced. Regarding residence, 36.4% (*n*=12/33) resided in the southern region, 27.3% (*n*=9/33) in the central region, 18.2% (*n*=6/33) in the eastern region, 12.1 (*n*=4/33) in the western region, and 6.1% (*n*=2/33) in the northern region.

Table 1 presents the tumour profiles of these patients. The primary site of the tumour was in the descended testis in 97% of cases (*n*=32/33). Both grade IV (undifferentiated anaplastic) tumours and distant metastasis were present in 45.5% (*n*=15/33) of patients. Tumours were distributed relatively equally between the left and right testicles (48.5% and 45.5%, respectively). Malignant tumour behaviour was detected in all 33 patients.

**TABLE 1:** Tumor profile (*n*=33).

	*n*	%
Primary site		
Descended testis	32	97
Undescended testis	1	3
Grade		
Grade I (well differentiated)	9	27.3
Grade II (moderately differentiated)	6	18.2
Grade III (poorly differentiated)	3	9.1
Grade IV (undifferentiated anaplastic)	15	45.5
Extension		
Localized	9	27.3
Regional: direct extension	6	18.2
Regional: lymph node and direct extension	3	9.1
Distant metastasis	15	45.5
Lateralization		
Right	15	45.5
Left	16	48.5
Bilateral	1	3
Base of diagnosis		
Histology of primary tumour	32	97
Histology of metastases	1	3

**Figure 1:**
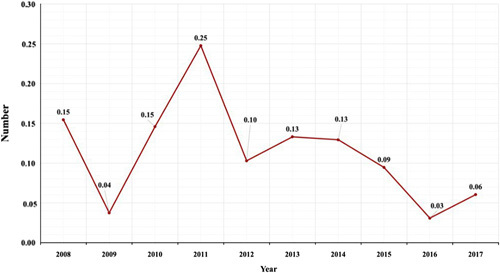
Incidence of choriocarcinoma combined with other germ cell elements per 1 000 000 throughout the year.

Figure 1 illustrates the incidence of mixed GCTs with choriocarcinoma components per 1 000 000 over the years, displaying an irregular pattern. The highest incidence rate was 0.25, observed in 2011, while the lowest incidence, 0.03, was observed in 2016. The overall mortality rate was 15% (*n*=5/33), with a mean time from diagnosis to death of 7.72 (SD=9) months, ranging from 0.5 to 21.5 months.

## Discussion

Leveraging data from the SCR between 2008 and 2017, we expanded upon the prior regional study by Mohammed Abomelha, which analyzed the SCR database from 1994 to 2013 and identified 1004 testicular cancer among Saudi adults^16^. A steady rise in the annual incidence of testicular cancer was observed, peaking at 94 cases in 2013^16^. Among his study population, 85.4% of testicular cancers were GCTs, with SGCTs constituting 40.7% and NSGCTs representing 44.6% of cases^16^. Among the NSGCTs, mixed GCTs (51.6%) were the most common, while pure choriocarcinoma was seen in 3.6% of patients^16^.

In terms of patient demographics, the mean age of our patients was 32 years, and 78.8% of cases occurred in individuals between 18 and 45 years, consistent with prior regional and Western data^10,16,18^. Li *et al.* recently demonstrated that an age older than or equal to 30 years is a negative predictor of outcomes in testicular choriocarcinoma patients, which may be an underlying reason for the advanced presentation and aggressive course of our patients^7^. Our results suggest an increasing testicular cancer incidence in Saudi Arabia, which echoes the findings of Abomelha’s study^16^. This trend is consistent with recent global data indicating a rise in testicular cancer incidence^19^. Nevertheless, we found the incidence of choriocarcinoma-containing mixed GCTs to be irregular, peaking in 2013 at 0.25.

Regarding tumour features, nearly all cases (32/33) of mixed GCTs with choriocarcinoma were unilateral without a specific predilection regarding laterality, in line with prior data^20^. Our results also corroborate previous studies suggesting that choriocarcinoma in mixed GCTs is a risk factor for an aggressive tumour phenotype characterized by high grade and metastasis at presentation^12,21^. Of the 1001 testicular cancer cases in our study, 3.3% were mixed GCTs with a choriocarcinoma component, of which almost half (45.5%) were grade IV anaplastic lesions and metastatic at the time of diagnosis, resulting in a mortality rate of 15% (5/33 patients). A study by Alvarado-Cabrero *et al.* observed a higher mortality rate than our findings, with all six patients with pure choriocarcinoma and five out of nine patients with choriocarcinoma-predominant mixed GCT succumbing to the disease during a median follow-up of 27 months^20^. To explain this, various risk factors may have differed between our studies, including the degree of b-HCG elevation and extrapulmonary metastases, recognized as poor prognostic factors in GCTs^22,23^. Interestingly, Hassan *et al.* observed that a small component of choriocarcinoma (<5%) in mixed GCTs does not exhibit the aggressive phenotype of pure choriocarcinoma or choriocarcinoma-predominant (>50%) mixed GCTs^24^. Hence, the degree of choriocarcinoma present within mixed GCTs may also explain discrepant findings on the outcomes of these tumours, perhaps related to a threshold amount of choriocarcinoma in the mixed GCT above which there is a poor prognosis^9^.

## Limitations

It is essential to acknowledge the limitations of our study. Potential under-reporting, a common challenge in case registries worldwide, may have affected the accuracy of our data. Additionally, the registry did not originally collect essential parameters such as potential risk factor exposure, sites of metastasis, mode of treatment, and socioeconomic status. Nevertheless, to the best of our knowledge, our study is the first to report population-based national data on mixed GCTs with choriocarcinoma components from Saudi Arabia. We believe that conducting more comprehensive chart reviews over longer periods will be crucial in developing improved strategies for early diagnosis and timely management of this disease.

## Conclusion

In summary, our findings align with reported data on choriocarcinoma-containing mixed GCTs, emphasizing their aggressive phenotype at presentation. Our study also provides a better understanding of this tumour’s epidemiological and clinicopathological aspects in Saudi Arabia. Our study sheds light on the clinical behaviour and outcomes of mixed germ cell tumours with choriocarcinoma components in Saudi Arabia. These tumours, while rare, tend to occur more frequently in young populations and present with an aggressive phenotype characterized by high-grade tumours and distant metastasis at diagnosis. The mortality rate among patients with mixed germ cell tumours containing choriocarcinoma was considerable, underscoring the poor prognosis associated with this malignancy. Future studies should focus on elucidating important prognostic factors to enhance the management of patients with these tumours.

## Ethical approval

The study was IRB approved (approval number NRC21/223/05). Patient confidentiality was ensured, and the patients' data were collected and used by the research team only. Serial numbers were used instead of medical record numbers to ensure anonymity. Due to the retrospective nature of the study, and the use of anonymized patient data, the requirement for informed consent was waived. Ethical approval (NRC21.223.05) was obtained from King Abdullah International Medical Research Center (KAIMRC) prior to the beginning of this study. This study is a secondary analysis.

## Consent

Not applicable.

## Sources of funding

This research did not receive any funding from institutions in the public, commercial, or not-for-profit sectors.

## Author contribution

All authors contributed to the research and/or preparation of the manuscript. M.A., A.S., and B.N.S. participated in the study design and wrote the first draft of the manuscript. A.A., Y.N., M.A., A.S., B.N.S., A.S.E., M.B., M.A.O., T.Z.A., S.A.R., B.A., R.A., M.A.L., and K.A. collected and processed the data. M.A. participated in the study design and performed the statistical analyses. M.A., A.S., and B.N.S. reviewed and finalized the manuscript. All authors read and approved the final manuscript.

## Conflicts of interest disclosure

The authors declare that there are no conflicts of interest.

## Research registration unique identifying number (UIN)

researchregistry9185.

## Guarantor

Mohammad Alghafees.

## Availability of data and material

Not applicable.

## Provenance and peer review

Not commissioned, externally peer-reviewed.

## Code availability

Not applicable.
